# Prognostic and predictive value of FCER1G in glioma outcomes and response to immunotherapy

**DOI:** 10.1186/s12935-021-01804-3

**Published:** 2021-02-12

**Authors:** Houshi Xu, Qingwei Zhu, Lan Tang, Junkun Jiang, Huiwen Yuan, Anke Zhang, Meiqing Lou

**Affiliations:** 1 Department of Neurosurgery, Shanghai General Hospital, Shanghai Jiao Tong University School of Medicine, Shanghai, 200080 China; 2 Department of Neurosurgery, Second Affiliated Hospital, School of Medicine, Zhejiang University, Zhejiang, 310029 China; 3 Tongji University, Shanghai, China

**Keywords:** Gliomas, Immunotherapy, T cell, FCER1G, biomarker

## Abstract

**Purpose:**

Glioma is the most prevalent malignant form of brain tumors, with a dismal prognosis. Currently, cancer immunotherapy has emerged as a revolutionary treatment for patients with advanced highly aggressive therapy-resistant tumors. However, there is no effective biomarker to reflect the response to immunotherapy in glioma patient so far. So we aim to assess the clinical predictive value of FCER1G in patients with glioma.

**Methods:**

The expression level and correlation between clinical prognosis and FER1G levels were analyzed with the data from CGGA, TCGA, and GEO database. Univariate and multivariate cox regression model was built to predict the prognosis of glioma patients with multiple factors. Then the correlation between FCER1G with immune cell infiltration and activation was analyzed. At last, we predict the immunotherapeutic response in both high and low FCER1G expression subgroups.

**Results:**

FCER1G was significantly higher in glioma with greater malignancy and predicted poor prognosis. In multivariate analysis, the hazard ratio of FCER1G expression (Low versus High) was 0.66 and 95 % CI is 0.54 to 0.79 (P < 0.001), whereas age (HR = 1.26, 95 % CI  1.04–1.52), grade (HR = 2.75, 95 % CI 2.06–3.68), tumor recurrence (HR = 2.17, 95 % CI  1.81–2.62), IDH mutant (HR = 2.46, 95 % CI 1.97–3.01) and chemotherapeutic status (HR = 1.4, 95 % CI  1.20–1.80) are also included. Furthermore, we illustrated that gene FCER1G stratified glioma cases into high and low FCER1G expression subgroups that demonstrated with distinct clinical outcomes and T cell activation. At last, we demonstrated that high FCER1G levels presented great immunotherapeutic response in glioma patients.

**Conclusions:**

This study demonstrated FCER1G as a novel predictor for clinical diagnosis, prognosis, and response to immunotherapy in glioma patient. Assess expression of FCER1G is a promising method to discover patients that may benefit from immunotherapy.

## Introduction

Glioma is served as the most prevalent malignant tumor in central nervous system, which accounts for more than 70 % of intracranial tumors with high degree of malignancy [1, 2]. Arising from glia cells, gliomas can be subdivided into a broad category of tumors, such as astrocytoma, oligodendroglioma, and glioblastoma (GBM). Regardless of tumor aggressiveness and malignancy, the average median time of overall survival is only 12–18 months [3, 4]. Although a variety of therapies are currently available, including surgery, radiotherapy, chemotherapy and immunotherapy, they still remain a low survival. Therapeutic response rely on intra-tumoral heterogeneity and intricacy programmed by genetic and epigenetic effectors. Besides, there are many physiological barriers, like blood-brain barrier (BBB), as a challenge to effective treatments. Driven by the infiltrative nature of gliomas, surgical resection seems to be an ineffective long-term procedure and recurrence often occur with fatal consequences. Moreover, aggressive therapies compromised the patient’s life quality and drives harmful side effects. Therefore, great understanding of the biological behavior and mechanism underlying tumor progression is essential to improve clinical diagnosis and therapeutic prognosis, even for the development of novel effective therapies.

Currently, cancer immunotherapy based on immune checkpoint blockades (ICBs), notably anti-CTLA4 (cytotoxic T-lymphocyte associated protein 4), anti-PDCD1/PD-1 (programmed cell death 1), anti-CD274/PD-L1, has emerged as a revolutionary treatment for patients with advanced highly aggressive therapy-resistant tumors. Unfortunately, the clinical reality is that only a small number of patients benefit from immunotherapy. Moreover, there is no effective biomarker to reflect the response to immunotherapy in glioma patient so far.

With the development of high-throughput microarray technology, gene expression profiles have been used to identify genes associated with progression and clinical prognosis of glioma [5–7]. A gene signature identified from four different published microarrays has been validated in GBM and LGG cohorts [8–10]. However, the predictive significance of the gene signature in glioma patients is unclear and is not currently applied in clinical practice. FCER1G is a key molecule involved in allergic reactions [11], located on chromosome 1q23.3 and encodes the γ subunit of fragment crystallizable (Fc) region (Fc R) of immunoglobulin. Fc R is a signal-transducing subunit that plays an critical role in chronic inflammatory programs. The binding between the Fc of immunoglobulins and the Fc R of immune cells activates cellular effector functions and may trigger destructive inflammation, immune cell activation, phagocytosis, oxidative burst, and cytokine release [12–14]. It has been illustrated that FCER1G participated in various diseases, such as squamous carcinogenesis, diabetic kidney disease, multiple myeloma, and clear cell renal cell carcinoma [12, 15–17]. However, the role of FCER1G in tumor progression and underlying molecular mechanisms are poorly understood. This study aimed to demonstrated FCER1G as a promising predictive target for glioma prognosis and response to immunotherapy.

## Materials and methods

### Tumor samples collection

Human glioma tissues were considered exempt by the Human Investigation Ethical Committee of Shanghai General Hospital affiliated to Shanghai Jiao Tong University. Human tumor samples were consecutively recruited between January 2019 and January 2020 from the Department of Neurosurgery in Shanghai General Hospital. A total of 20 patients with glioma underwent the surgery for the first time and had not previously received radiotherapy or chemotherapy.

### Data source and expression analysis

Pan-cancer dataset in The Cancer Genome Altas (TCGA) which consists of 33 kinds of cancer and adjacent tissue samples or GTEx expression matrixs were analyzed with UCSCXenaShiny [18] (https://hiplot.com.cn/advance/ucsc-xena-shiny). In this study, we analyzed both GBM and LGG. All the glioma datasets and were obtained from Gliovis [19] (http://gliovis.bioinfo.cnio.es/), including six datasets containing 2336 samples : 642 grade II patients, 780 grade III patients and 914 grade IV patients. (Additional  file 1: Table S1)

### **Immunohistochemical analysis** 

Patient tumor samples were fixed in 4 % paraformaldehyde for 24 hours and then embedded in paraffin. Paraffin blocks were cut into 5 µm sections. Rehydrated tissue sections were blocked with 5 % BSA overnight at 4 ℃ and then were stained with FCER1G (Abcam, ab151986, USA). After washing with PBS, the sections were incubated with biotinylated anti-rabbit IgG (Vector Laboratories, CA, USA). The ABC method (Vector Laboratories) was used. The sections were observed using an AX-80 microscope (Olympus, Tokyo, Japan). Images were dealt with Image J software and relative expression was calculated.

### Real‐time PCR

Total RNA was extracted from human tumor tissues using TRIzol reagent (Invitrogen, Carlsbad, CA, USA) and reverse transcripted using FastQuant RT kit (Tiangen, Shanghai, China). Real-time PCR was carried out using SuperReal SYBR Green kit (Tiangen, Shanghai, China) and Lightcycler 96 (Roche, Penzberg, Germany). The primer sequences were listed as follow: FCER1G forward: GCCTGCATGCCATTAACACC; reverse: AACAGGGAGGAGGAACCACT; PDCD1 forward: CAGTTCCAAACCCTGGTGGT; reverse: GGCTCCTATTGTCCCTCGTG.

### **Immune cells and bioinformatic analysis** 

The single sample gene set enrichment analysis (ssGSEA) was used to define a enrichment score to represent the degree of absolute enrichment of a gene set in each sample within a given dataset with R package “*GSVA*” [20]. Normalized enrichment scores could be calculated for each immune category. 28 types of immune cells’ gene set signatures were obtained from a previous study [21]. (Additional  file 1)

Based on the median expression values of FCER1G, CGGA dataset was divided into a high FCER1G expression group (top 50 %) and a low FCER1G expression group (bottom 50 %). R package “*limma*” was used for differential expressed gene (DEGs) analysis. The biological significance of the DEGs was defined as |logFC|≥1.5 and adj.pvalue < 0.05. Gene Ontology (GO) including biological process (BP), molecular function (MF) and cellular component (CC) and Kyoto Encyclopedia of Genes and Genomes (KEGG) analyses were utilized for gene set annotation using the R package “*clusterProfiler*” [22]. Gene Set Enrichment Analysis (GSEA) was further used to investigate the functional enrichment with R package “*Pi*“ [23]. To explore the correlation between the expression levels of FCER1G and immune status, a total of 25 immunity- related gene sets covering both innate and adaptive responses were from a previous study [24] (Additional  file 1). Gene Set Variation Analysis from R package GSVA [20] was performed to obtain the immune profile of the glioma samples.

### **Quantify of relative abundance of TIICs and prediction of the immunotherapy response** 

The CGGA dataset (n = 1013, Grade II = 291, Grade III = 334 and Grade IV = 388) was used as the discovery set and the TCGA-GBMLGG dataset(n = 620, Grade II = 226, Grade III = 244 and Grade IV = 150) was used as the validation set. Immune Cell Abundance Identifier (ImmuCellAI) [25] (http://bioinfo.life.hust.edu.cn/ImmuCellAI#!/analysis) is a novel algorithm that uses gene set signatures to estimate the abundance of 24 immune cells from transcriptomic data. In contrast to other known algorithms designed to estimate immune cell composition from transcriptomic data, it focuses on subsets of T cells that are associated with tumor progression and initiation. The gene set signatures of the T-cell subsets used in this study are listed in the Supplementary Material ,which included 18 subtypes of T cells and 6 other types of immune cells. Moreover, ImmuCellAI can be used to predict the reponse of Immune checkpoint blockade (ICB) therapy with the ICB response prediction being checked.

To predict their putative response to anti-PDL1 drug, glioma samples were scored with the GSVA method using the T-cell inflammatory (TIS) signature. This signature was derived from a previous study [24] and listed in Additional  file 1.

Tumor immune dysfunction and exclusion (TIDE) (http://tide.dfci.harvard.edu/login/) is a computational method developed to predict the immune checkpoint blockade response based on pretreatment tumor gene profiles that integrate the expression signatures of T-cell dysfunction and T-cell exclusion to model the mechanisms of tumor immune evasion [26]. Furthermore, the Subclass Mapping (SubMap) method was applied to evaluate the expression similarity between the two subgroups and the patients with different immunotherapy responses [27]. P-values were used to evaluate the similarity, and the lower the P-values were, the higher the similarity. In this study, we utilized TIDE, TIS, SubMap and ImmuCellAI to predict the potential immunotherapy responses of patients with gliomas.

### Statistical analysis

All statistical analysis were carried out by R software 3.6.1. Kolmogorov-Smirnov tests were used to evaluate the distribution normality of each dataset to determine whether a non-parametric rank-based analysis or a parametric analysis should be utilized. Spearman correlation analysis were used for correlation analysis. The Fisher exact test and Wilcoxon rank-sum tests were used to test hypotheses in categorical and continuous variables, respectively. In the survival analysis, associations between characteristics and overall survival were evaluated by Cox proportional hazard models. Kaplan-Meier survival curves were drawn and compared among subgroups using log-rank tests with R packages “survival” and “survminer”. Meta-analysis was performed with R package “meta”. ROC curves, sensitivity as well as specificity were generated using R package “pROC”. For all statistical analyses, P value < 0.05 was considered significant.

## Results

### Pan-cancer analysis of FCER1G expression

**Fig. 1:**
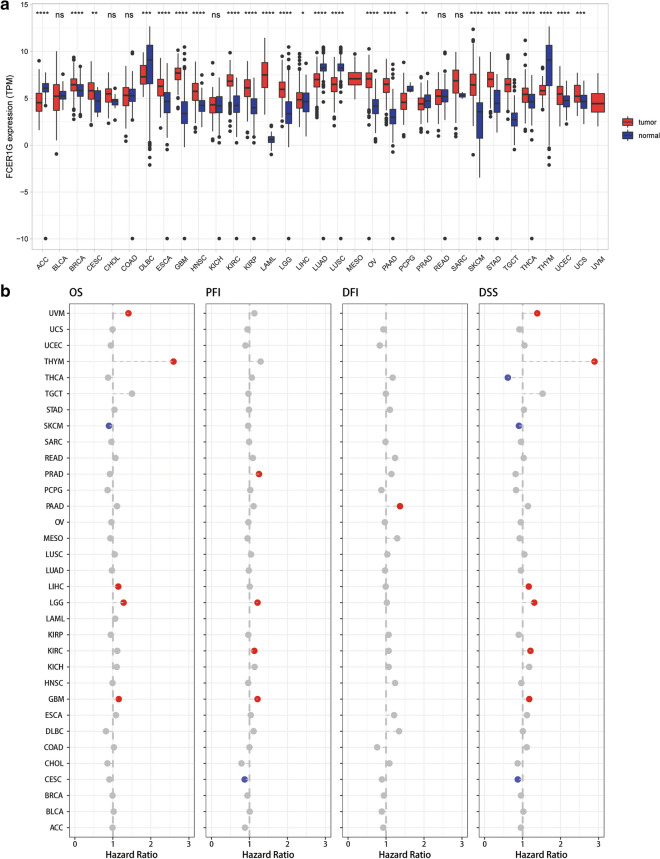
Pan-cancer analysis of FCER1G expression. **a** UCSCxena shiny was used to visualize FCER1G mRNA expression in The Cancer Genome Atlas (TCGA) pan-cancer datasets. *P < 0.05; **P < 0.01; ***P < 0.001; ****P < 0.0001, ns no significance (Wilcoxon test). **b** Dot plot of correlation between FCER1G with OS, PFI, DFI, DSS. (Red represents HR > 1 and P value < 0.05; Blue represents HR < 1 and P value < 0.05; Gray represents P value > 0.05)

Pan-cancer analysis showed a significant expression difference of FCER1G levels between a variety of tumors and adjacent tissues (or GTEx) (Fig. 1a and Additional file 2:  Fig. 1a). Expression of FCER1G was higher in BRCA, ESCA, GBM, HNSC, KIRC, KIRP, LAML, LGG, LIHC, OV, PAAD, SKCM, STAD, TGCT, THCA, UCEC, and UCS (p < 0.05) than normal tissues, while FCER1G was lower in tumor of ACC, DLBC, LUAD, LUSC, PRAD, and THYM (p < 0.05).

Patients in 33 types of tumor cohorts were then divided into high and low expressed group according to the median value of FCER1G gene expression. Subsequent survival analysis obtained significant differences across several cancer types. Specifically speaking, patients with high expression level of FCER1G showed a shorter overall survival (OS), progression-free interval (PFI) and disease-specific survival (DSS) than low expression patients both in LGG and GBM cohort (Fig. 1b).

### **The expression level of FCER1G increased with the progression of glioma** 

**Fig. 2:**
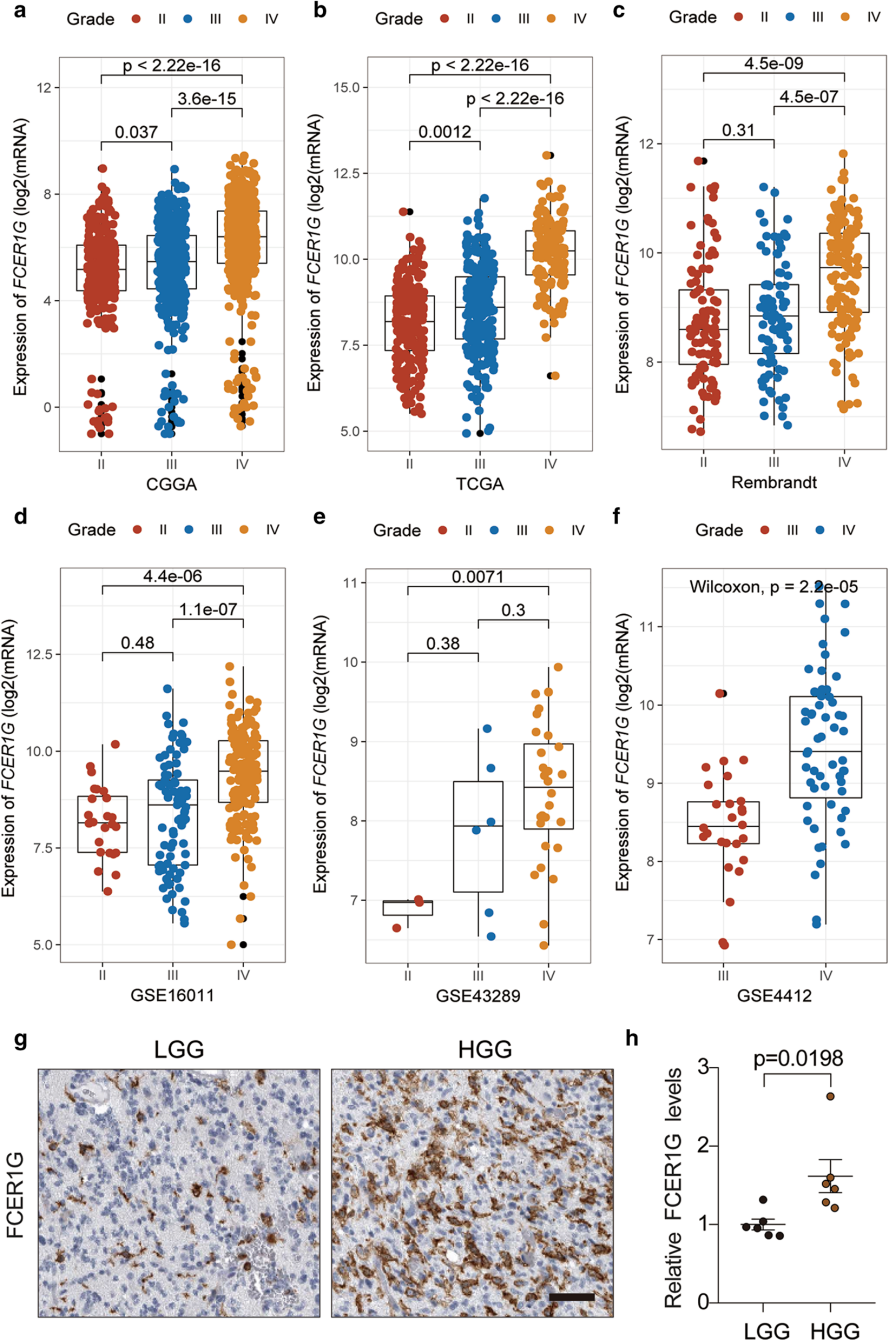
The expression level of FCER1G increased with the progression of glioma. The X-axis represents the WHO grade while the Y-axis represents FCER1G expression value(log2). Based on Wilcoxon test. **a** CGGA, **b** TCGA, **c** Rembrandt, **d** GSE16011, **e** GSE43289, and **f** GSE4412. **g** Representations and **h** quantification of immunohistochemistry detection of FCER1G in low grade glioma LGG and HGG

In the subsequent study, we focused on exploring the clinical value of FCER1G in gliomas. To explore the expression levels of FCER1G mRNA in different stages of gliomas, we used six datasets to analyze FCER1G expression levels. We observed that the expression level of FCER1G increased in glioma with high malignancy. In CGGA dataset, a significant increase of FCER1G expression was noted in WHO grade III (n = 334), and grade IV (n = 388) than grade II (n = 291) (IV versus III: P < 0.001; IV versus II: P < 0.001; III versus III: P = 0.037, Fig. 2a). In the TCGA-GBMLGG dataset, a remarkable upward trend in FCER1G expression with tumor progression was further confirmed in grade II (n = 226), III (n = 244) and IV (n = 150 glioma patients (IV versus III: P < 0.001; IV versus II: P < 0.001; III versus III: P = 0.0012, Fig. 2b). Furthermore, the same trend was also found in the Rembrandt dataset with 98 grade II, 85 grade III, and 130 grade IV patients (IV versus III: P < 0.001; IV versus II: P < 0.001; III versus III: P = 0.31, Fig. 2c). Moreover, according to analysis of GEO dataset, we also found that the GSE16011 cohort with grade II (n = 24), grade III (n = 85), and grade IV (n = 159) glioma patients (IV versus III: P < 0.001; IV versus II: P < 0.001; III versus III: P = 0.48, Fig. 2d), GSE43289 dataset with 3 grade II, 6 grade III, and 28 grade IV patients (IV versus III: P = 0.3; IV versus II: P = 0.0071; III versus III: P = 0.38, Fig. 2e), and the GSE4412 dataset (26 grade III and 59 grade IV patients, P < 0.0001, Fig. 2f) all exerted higher expression of FCER1G in high grade glioma.

To further validate these results, IHC for FCER1G and qRT-PCR was performed to assess FCER1G expression in patient-derived glioma tissue samples. As expected, in comparison with low grade glioma (LGG) tissues, a significant increase of FCER1G was revealed in high grade glioma (HGG) tissues (Figure. 2g, h). according to the above data, the expression of FCER1G increased with the development of glioma, suggesting that FCER1G may be involved in the malignant progression of glioma.

### **Increased FCER1G expression predicts poor prognosis in gliomas** 

After we illustrated the correlation between FCER1G expression level and tumor progression of glioma, we next investigated the prognostic value of FCER1G.

**Fig. 3:**
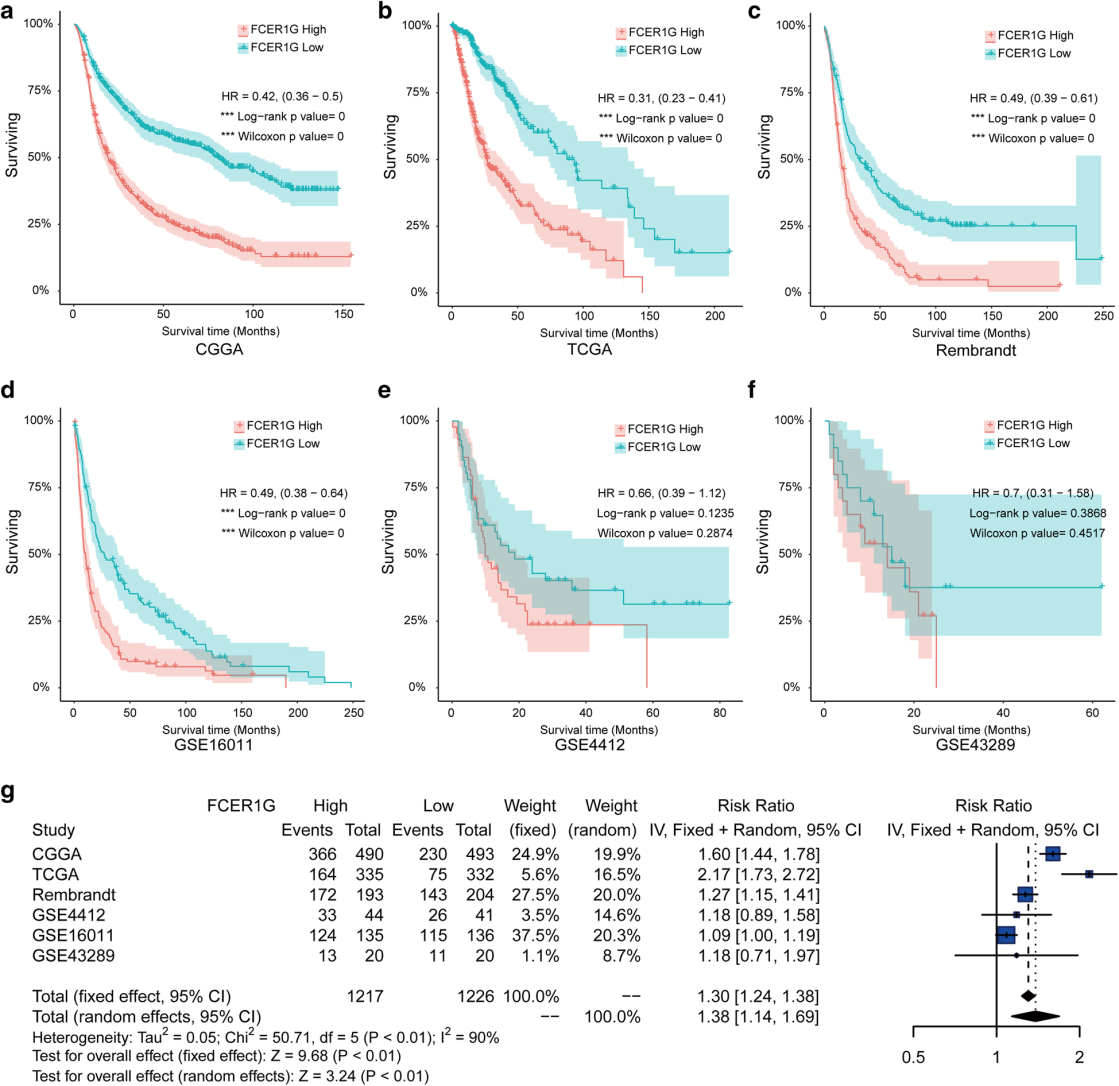
Increased FCER1G expression predicts poor prognosis in gliomas. Kaplan-Meier plots of FCER1G in a variety glioma datasets, 95 % CI (confidence interval) were also showed. Patients were divided into high and low expressed group by the medium expression level. **a** CGGA, **b** TCGA, **c** Rembrandt, **d** GSE4412, **e** GSE43289, and **f** GSE16011. **g** Forest plot of the RRs for patients with high FCER1G expression compared to patients with low FCER1G levels

According to the median value of FCER1G expression, patients were divide into high and low expression group. The Kaplan–Meier curve and log-rank test analysis revealed that patients with higher expression of FCER1G from CGGA (HR:0.69, 95 % CI 0.49–0.98), TCGA dataset (HR:0.31, 95 % CI 0.23–0.41), Rembrandt (HR:0.49, 95 % CI 0.39–0.61), and GSE16011 (HR:0.49, 95 % CI 0.38–0.64), showed significantly poorer overall survival (OS) than those with low expression (Fig. 3a, c, e and f), while patients from GSE43289 and GSE4412 dataset showed similar trend with no statistic significance (Fig. 3e, f). The sample sizes of the six cohorts were very different, three over 500 samples and two less than 200 samples. To improve the stability of the results, a fixed effects model was employed to pool the HRs of the six cohorts, and the result also validated that patients with high level of FCER1G expression exerted shorter OS times than patients with low expression level (RR = 1.30, 95 % CI  1.24–1.38, Fig. 3g).

To better understand the role of expression of FCER1G in patients with glioma, we analyzed the CGGA dataset with clinical data of 1013 glioma patients. We divided the patients into high expression group (n = 506) and low expression group (n = 507) based on FCER1G levels. Through univariate analysis of clinical characteristics, we found that FCER1G was more likely to be associated with older age (P = 0.002), high malignancy (P < 0.001), GBM type (P < 0.001), post-operative relapse (P < 0.001), poorer survival (P < 0.001), IDH wild type (P < 0.001), and different therapeutic options (Radiotherapy, P = 0.047; chemotherapy, P = 0.009), however, there is no significant differences in gender (Table 1).

**Table 1:** Clinical characteristics of 1013 glioma patients in the CGGA dataset according to FCER1G expression levels

FCER1G expression	High	Low	P value
n	506	507	
FCER1G_mRNA (median [IQR])	6.74 [6.22, 7.42]	4.69 [3.81, 5.22]	< 0.001
Age (median [IQR])	44.00 [36.00, 54.00]	41.00 [34.00, 49.00]	0.002
Gender (%)			0.371
Female	199 (39.3)	214 (42.2)	
Male	307 (60.7)	293 (57.8)	
Grade (%)			< 0.001
II	101 (20.0)	187 (36.9)	
III	140 (27.7)	193 (38.1)	
IV	261 (51.6)	126 (24.9)	
	4 ( 0.8)	1 ( 0.2)	
Histology (%)			
Anaplastic Astrocytoma	118 (23.3)	95 (18.7)	
Anaplastic Oligoastrocytoma	2 ( 0.4)	19 ( 3.7)	
Anaplastic Oligodendroglioma	19 ( 3.8)	75 (14.8)	
Astrocytoma	81 (16.0)	92 (18.1)	
GBM	261 (51.6)	126 (24.9)	
Oligoastrocytoma	2 ( 0.4)	7 ( 1.4)	
Oligodendroglioma	19 ( 3.8)	92 (18.1)	
	4 ( 0.8)	1 ( 0.2)	
Recurrence (%)			0.001
Primary	296 (58.5)	350 (69.0)	
Recurrent	186 (36.8)	147 (29.0)	
Secondary	20 ( 4.0)	10 ( 2.0)	
	4 ( 0.8)	0 ( 0.0)	
Subtype (%)			< 0.001
Classical	110 (21.7)	52 (10.3)	
Mesenchymal	95 (18.8)	19 ( 3.7)	
Proneural	82 (16.2)	74 (14.6)	
	219 (43.3)	362 (71.4)	
survival (median [IQR])	17.50 [8.80, 40.60]	37.00 [15.35, 75.85]	< 0.001
status (%)			
Alive	125 (25.3)	260 (53.1)	< 0.001
Dead	369 (74.7)	230 (46.9)	
IDH status (%)			< 0.001
Mutant	213 (42.1)	316 (62.3)	
Wildtype	287 (56.7)	145 (28.6)	
	6 ( 1.2)	46 ( 9.1)	
1p19q (%)			< 0.001
Codel	39 ( 7.7)	172 (33.9)	
Non-codel	461 (91.1)	263 (51.9)	
	6 ( 1.2)	72 (14.2)	
Radio status (%)	68 (14.9)	94 (20.0)	0.047
No			
Yes	388 (85.1)	376 (80.0)	
Chemo status (%)	117 (26.1)	156 (34.2)	0.009
No			
Yes	332 (73.9)	300 (65.8)	

By using the Cox regression model, we computed multivariate hazard ratios for different variables of 1013 glioma patients. In multivariate analysis, the hazard ratio of FCER1G expression (Low versus High) was 0.66 and 95 % CI is 0.54 to 0.79 (P < 0.001), whereas age (HR = 1.26, 95 % CI  1.04–1.52), grade (HR = 2.75, 95 % CI 2.06–3.68), tumor recurrence (HR = 2.17, 95 % CI  1.81–2.62), and chemotherapeutic status (HR = 1.4, 95 % CI  1.20–1.80) are also included (Table 2). The expression level of FCER1G was significantly related to the OS in glioma patients. FCER1G expression value was a stable factor affecting the survival level of glioma patients.

**Table 2:** Univariate and multivariate analysis for overall survival of glioma patients

Variable		Univariate analysis			Multivariate analysis		
Age		HR	95 % CI	pvalue	HR	95 % CI	pvalue
(≥ 40 vs. < 40)		1.6	(1.4-2.0)	< 0.001	1.26	(1.04 − 1.52)	0.017
Gender							
Female vs. male		0.98	(0.83–1.2)	0.79			
Grade							
II vs. III vs. IV		3.6	(2.2–6.2)	< 0.001	2.75	(2.06 − 3.68)	< 0.001
Recurrence							
Primary vs. Recurrent vs. Secondary		2.5	(1.8–3.2)	< 0.001	2.17	(1.81 − 2.62)	< 0.001
IDH status							
Wildtype vs. Mutant		3.1	(2.6–3.6)	< 0.001	2.46	(1.97–3.01)	< 0.001
Radio status							
Yes vs. no		1	(0.83–1.3)	0.73			
Chemo status							
Yes vs. no		1.5	(1.3–1.9)	< 0.001	1.4	(1.2–1.8)	< 0.001
FCER1G							
Low vs. High		0.43	(0.36–0.51)	< 0.001	0.66	(0.54 − 0.79)	< 0.001

### FCER1G is associated with immune infiltration and immune activation in gliomas

**Fig. 4:**
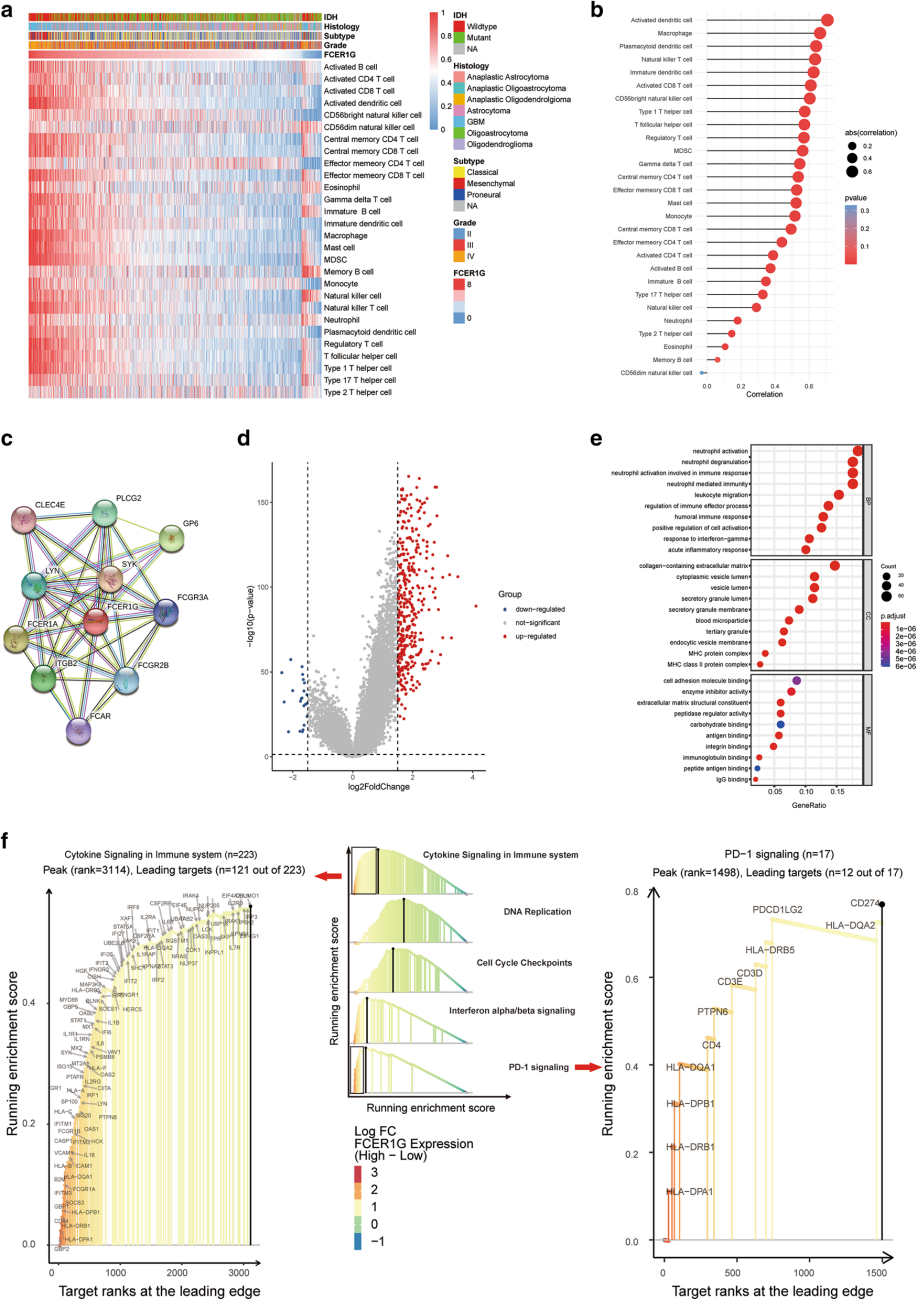
FCER1G is associated with immune infiltration and immune activation in gliomas. **a** Heatmap showing FCER1G-associated relative abundance of 28 immune cells in gliomas, annotations show corresponding clinical features of each sample. **b** The correlation between the ssGSEA scores of 28 immune cells and the expression of FCER1G in gliomas. **c** STRING database shows the PPI network of FCER1G. **d** Volcano plot of the DEGs expression between FCER1G high and FCER1G low. Cut-off criteria for DEGs significance was adj. p value < 0.05 and the absolute value of the log2 fold change > 1.5. The Y-axis displays the -log10 P-value for each gene, while the X-axis displays the log2 fold change for that gene relative to FCER1G expression. e GO results for differential expression genes. The X-axis represents gene ratio and the Y-axis represents different enriched pathways (BP: biological progress; *CC* cellular component, *MF* molecular function). f Rank-based gene set enrichment analysis shows significantly activated immune related pathways in FCER1G high subgroup compared with FCER1G low (LFC, log fold change)

Patients diagnosed with the same histological cancer types may have different immune infiltration levels, which could lead to diverse clinical outcomes. The immune profile of gliomas relating to the prognosis and immunotherapy has been widely reported in several cancers, including gliomas. FCER1G is served as an important regulatory player, involving in initiating the transfer from T-cells to the effector T-helper 2 type and mediating the allergic inflammatory signaling of mast cells and interleukin 4 production from basophils [28, 29]. Therefore, the correlation of FCER1G and immune infiltration levels was evaluated to reveal the possible mechanism by which FCER1G affects the prognosis of gliomas. The relative quantity of the 28 immune cells from the CGGA dataset was systematically estimated using the ssGSEA algorithm (Fig. 4a). The correlations of FCER1G expression with infiltrating levels of immune cells was evaluated by spearman method, which revealed close relationship between FCER1G with T cells, macrophages, and B cells (Fig. 4b). These results suggested that FCER1G expression was involved in immune infiltration remodeling of gliomas.

Next, we try to further elucidate the relationship between FCER1G expression and immune infiltration and to explore the molecular mechanisms of FCER1G with STRING database. The result showed that FCER1G had a closely interactions with FCGR3A, ITGB2, LYN, SYK, in which FCER1G acts as a core gene (Fig. 4c). Moreover, we analyzed the differential expression values between high and low FCER1G group. A total of 372 genes were up-regulated and 22 genes were down-regulated (adj.pvalue < 0.05, FC > 1.5 or <-1.5, Fig. 4d).

Then we analyzed the enriched GO terms and KEGG pathways with the DEGs. Among the biological process terms of GO, most of DEGs were enriched in neutrophil activation, leukocyte migration, collagen-containing extracellular matrix, and cell adhesion molecule binding (Fig. 4e). According to the KEGG analysis results, staphylococcus aureus infection, phagosome, and cell adhesion molecules (CAMs) were remarkably enriched (Additional file 3:  Fig. S2).

Gene set enrichment analysis (GSEA) was also used to explore the mechanisms of FCER1G in gliomas. The CGGA data were analyzed with “MsigdbC2KEGG” (KEGG gene set, listed in Additional file 1). The enrichment results (nominal p value < 0.05 and FDR < 0.25) are shown in Additional file 1: Sheet 3. Results showed that various immune activation and tumor progression associated genes were enriched, especially in cytokine signaling in immune, DNA replication and PD-1 signaling (Fig. 4f), reflecting relatively enhanced tumor progression and activated inflammation.

### **Identification of the correlation between FCER1G and immune phenotype of gliomas** 

**Fig. 5:**
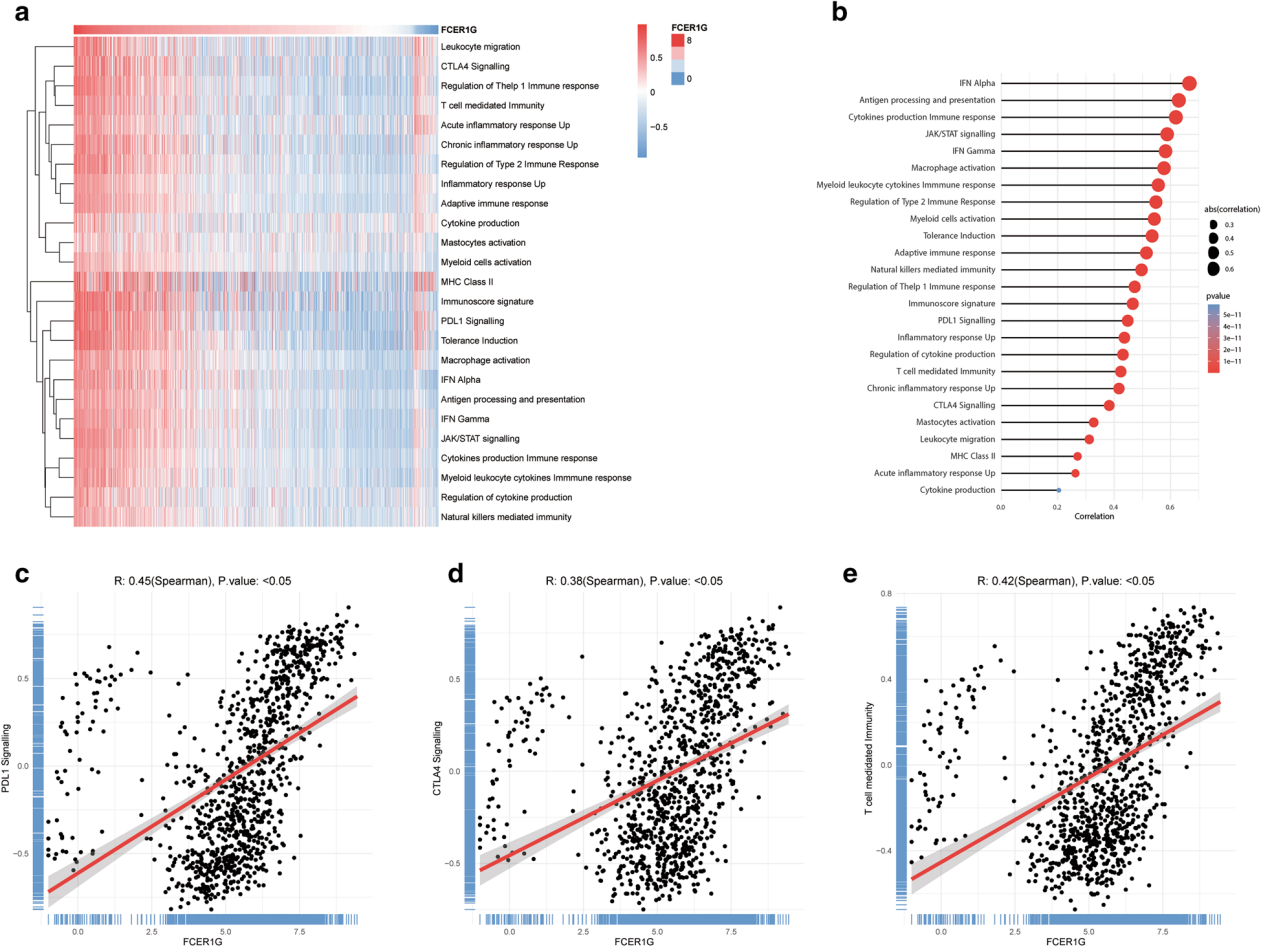
Identification of the correlation between FCER1G and immune phenotype of gliomas. **a** Heatmap showing FCER1G-associated GSVA scores of 25 innate and adaptive immunity-related gene sets. **b** The correlation between the GSVA scores of 25 innate and adaptive immunity-related gene sets and the expression of FCER1G in gliomas. **c **The correlation between the PDL1 signaling and the expression of FCER1G. **d** The correlation between the CTLA4 signaling and the expression of FCER1G. **e** The correlation between the T cell mediated immunity and the expression of FCER1G

To further explore the existence of malignant gliomas with a hot immune phenotype, manually curated gene sets related to both adaptive and innate immune responses were used to quantify the immune phenotype (Fig. 5a). The heatmap showed that, with increasing FCER1G expression, the immune phenotype tended to be “hot”. This was consistent with the conclusions drawn above that FCER1G played a key role in the glioma activated immune response. The Spearman’s test revealed a high correlation between the expression of FCER1G with PDL1 signaling (r = 0.45, P < 0.05), CTLA4 signaling (r = 0.38, P < 0.05), and T cell mediated immunity (r = 0.42, P < 0.05), which further confirmed the findings in GSEA results (Fig. 5b, d).

### **Subgroups divided by FCER1G expression predict potential immunotherapy responses of gliomas** 

**Fig. 6:**
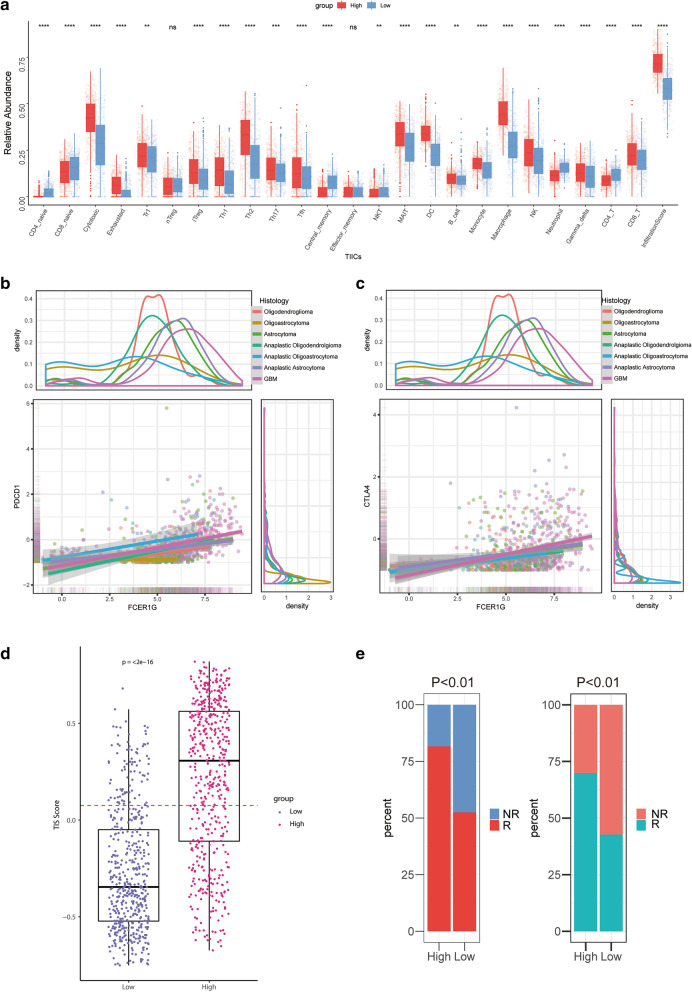
Subgroups divided by FCER1G expression predict potential immunotherapy responses of gliomas. **a** The fraction of TILCs in FCER1G high and low subgroups. Within each group, the scattered dots represent TILCs expression values. The thick line represents the median value. The bottom and top of the boxes are the 25th and 75th percentiles, interquartile range. The whiskers encompass 1.5 times the interquartile range. The statistical difference of three gene clusters was compared through the Kruskal–Wallis test. **b** The correlation between the expression of FCER1G and PDCD1 (**b**) and CTLA4 **c** in CGGA cohort. **d** T-cell inflammatory signature (TIS) scores across FCER1G subgroups. A plot presents a single glioma sample. Red line indicates the median value. **e** Rates of the different anti-PD1 and anti-CTLA4 responses of patients from the CGGA cohort predicted by the ImmunCellAI (Left, Chi-square test, P < 0.01) and Tumor Immune Dysfunction and Exclusion (TIDE) web program (Right, Chi-square test, P < 0.01) in the high or low FCER1G subgroups. *P < 0.05; **P < 0.01; ***P < 0.001; ****P < 0.0001

The above findings suggested that FCER1G was closely associated with T cells, which play an important role in immunosurveillance evasion in malignant gliomas [30]. Strong correlations were found between PD1 (PDCD1) and PDL1 (CD274)/PDL2 (PDCD1LG2), between CTLA4 and CD80/CD86, and between CXCR4 and CXCL12 in gliomas Additional file 4: Fig. 3a-c). The relative abundances of 24 types of immune cells in the TME of gliomas were quantified with ImmuCellAI. Notably, the proportions of TIICs showed marked variations between the FCER1G high and low subgroups (Fig. 6a). Moreover, FCER1G showed significant correlations with PD1 (r = 0.42, P < 0.01), PDL1 (r = 0.62, P < 0.01),and CTLA4 (r = 0.34, P < 0.01) (Fig. 6b, c), same conclusions were also drawn in analysis of TCGA GBMLGG dataset (Additional file 4: Fig. 3d, e). To verify transcriptome results from public datasets, 20 patients from Shanghai general hospital were included in our study and quantitative real-time PCR were utilized to investigated the correlation between expression levels of FCER1G and PD1, and the results showed that FCER1G was positively correlated with PD1 (r = 0.62, p < 0.01) (Additional file 5: Fig. 4a). Patients with high FCER1G expression showed high levels of the therapeutic targets PD1, PDL1 and CTLA4, which indicated a hypothetic treatment as immune checkpoint.

To further validate this hypothesis, we utilized T cell inflammatory signature (TIS) scores in high and low FCER1G subgroups. Patients with high FCER1G expression get higher scores in the TIS signature (P < 0.001), reporting to be correlated with response to anti PDL1 checkpoint inhibitor pembrolizumab, which supporting the hypothesis (Fig. 6d). Furthermore, the possibility of immunotherapy response was predicted in patients with gliomas by ImmuCellAI and TIDE algorithm. The ImmuCellAI predicted that patients with high FCER1G levels were more likely to respond to immunotherapy (81.6 %, 413/506, CGGA) than low FCER1G subgroup (52.5 %, 266/507, CGGA. Figure 6e). Similar findings were obtained in the validation set, with high predictive efficacy of FCER1G for immunotherapy response in glioma patients (AUC: CGGA 72.11 % (69.83–74.92 %), TCGA 71.73 % (68.96–73.44 %). Additional  file 5: Fig. 4c, d), as well as high sensitivity and specificity (CGGA(sensitivity = 61.38 %, specificity = 78.98 %), TCGA(sensitivity = 60.36 %, specificity = 79.11 %)). Meanwhile, TIDE also suggested that high levels of FCER1G tended to more likely respond to immunotherapy (69.0 %, 349/506, CGGA) than low FCER1G subgroup (41.8 %, 212/507, CGGA. Figure 6e). We also utilized the submap algorithm [27] to compare the similarity of the expression profiles between the two subgroups of glioma patients and 47 previous melanoma patients with detailed immunotherapeutic information, and revealed that patients in FCER1G-high subgroup were more responsive to anti-PD1 treatment (Bonferroni corrected P value = 0.008) (Additional file 5 Fig. 4b), which was consistent with the previous conclusions (Additional file 6: Fig. 5).

Taken together, FCER1G may be a good index for quantifying the tumor immune microenvironment and prediction for immunotherapy responses of gliomas.

## Discussion

FCER1G, known as FcRγ, is a key molecule involved in tumor progression. Previous studies have shown that is an innate immunity gene and involved in the development of eczema, clear cell renal cell carcinoma, meningioma, and childhood leukemia [17, 31–33]. In our study, great malignancy and poor outcomes have been confirmed in patients in FCER1G-high group compared to the FCER1G-low group. to gain insight into intrinsic mechanism and signal pathways, DEGs between the two group were analyzed. As a result, up-regulated DEGs in the subgroup with poor outcomes are enriched in immune response and inflammatory response, which was also confirmed by both KEGG functional enrichment analysis and GSEA analysis. Tumor progression is a complex process that requires interaction between cancer cells, the microenvironment, and the immune system, influencing both tumor initiation and progression [34]. Recent research suggests that immune system cells have an essential accessory role of preserving tissue integrity and function during homeostasis, infection, and noninfectious perturbations by eliminating pathogens, exerting some influence on the clinical outcomes of tumors [35, 36]. Many studies have also demonstrated that high immune infiltration is associated with improved clinical outcomes and better response to treatment in cancers [37–42]. We illustrated that various immune activation and tumor progression associated genes were enriched, especially in cytokine signaling in immune, DNA replication and PD-1 signaling by GSEA. The cytokine signaling and PD-1 signaling pathways have been identified as key signaling pathways in immunotherapy to glioma.

In this study, a cox regression model was built to predict the prognosis of glioma patients with multiple factors, including FCER1G expression, age, grade, tumor recurrence, IDH status, and chemotherapeutic status. Furthermore, we illustrated gene FCER1G as a novel diagnostic and therapeutic target for the first time, which stratified glioma cases into high and low FCER1G expression subgroups that demonstrated with distinct clinical outcomes. Then, we explore the underlying molecular mechanisms of FCER1G in tumor progression and potential correlation between FCER1G expression and immune cell activation and response to immunotherapy in patients with glioma.

The treatment of gliomas is highly individualized and tests are available to guide the use of radiotherapy or chemotherapy. In instance, O [6]-methylguanine-DNA methyltransferase (MGMT) testing assesses drug resistance in temozolomide-based chemotherapy [43, 44]. Besides, radio-sensitivity and XPO1 expression were combined to predict the effectiveness of radiotherapy [45]. However, there is a lack of a diagnostic biomarker guiding adjuvant immunotherapy, in which immune checkpoint is a possible factor.

Currently, the clinical benefit of ICB is only observed in a minority of patient with glioma, many of which tend to relapse after a short-term benefit. The type, density, functionality, and location of different immune cell in the tumor microenvironment are major factors predicting the response to ICB. Indeed, tumor infiltrated with preexisting T cells are more likely to present response to ICBs. Thus, majority of tumors can be defined as “cold” immune desert tumors and “hot” inflamed immune infiltrated tumors [46, 47]. In line with this concept, it is a novel strategies to explore biomarker to assess tumor immune microenvironment and predict tumor sensitivity to immunotherapy. Our research, with large sample size of 1013 patients, confirmed that the FCER1G is a novel independent prognostic predictor to find patients who respond to immunotherapy effectively.

The relative abundances of 24 types of immune cells in the TME of gliomas were quantified with ImmuCellAI. Notably, patients with high FCER1G expression showed high levels of the therapeutic targets PDL1 and CTLA4, which indicated a hypothetic treatment as immune checkpoint. PDL1 is a key negative regulator for immune inhibitory axis signaling controlling T lymphocyte infiltration in solid tumors, which is widely expressed in glioma cell lines [48, 49] and human specimens [50, 51]. PD-L1 is recently served as a oncogenic gene. Down-regulation of PDL1 significantly decreases tumor volume of U87 glioma in nude mice, while over-expression of PDL1 promotes tumor progression [52]. Moreover, CTLA4 is one of the most fundamental immunosuppressive cytokines, which inhibits T-cell activation and terminates the T-cell response [53]. Positive correlation between FCER1G with PD-L1 and CTLA4 indicated its predictive value in response to immunotherapy. Furthermore, patients in FCER1G-high subgroups get higher TIS scores, reporting to be correlated with response to anti PDL1 checkpoint inhibitor pembrolizumab. The possibility of immunotherapy response was predicted in patients with gliomas by ImmuCellAI, SubMap and TIDE algorithm, both of which suggested that high levels of FCER1G tended to more likely respond to immunotherapy.

Despite these findings, there is a limitation for this study exist. The data of samples were download from CGGA, TCGA, and GEO database and the particular information about the extent of surgical resection was not provided, which is a critical factor for overall survival. Thus, further analysis with more detailed clinical information should be presented in following studies. And we lack sufficient clinical data to validate the predictive value of FCER1G for glioma immunotherapy response, we will continue to investigate the potential predictive value of FCER1G in future studies.

In summary, this study demonstrated FCER1G as a novel predictor for clinical diagnosis, prognosis, and response to immunotherapy in glioma patients. Assess expression of FCER1G is a promising method to discover patients that may benefit from immunotherapy. These results are of great clinical significance and will contribute to personalized therapy.

## Supplementary Information

** Additional file 1:** Sheet 1. Summary of datasets used in the study. Sheet 2. Marker genes of 28 immune cells. Sheet 3. Marker genes of TILCs. Sheet 4. Marker genes of TIS score. Sheet 5. GSEA result of FCER1G high vs low subgroups.

** Additional file 2: Figure S1.** FCER1G expression and correlation with immune signatures in pan-cancer. **a** Anatomy graph shows significant differences in expression levels of FCER1G in various tumors and normal tissues. **b** FCER1G has a significant relationship with tumor microenvironment in various tumors.

** Additional file 3: Figure S2.** Immune cell fraction and GO analysis in FCER1G high and low subgroups. **a** The fraction of 28 immune cells in FCER1G high and low subgroups. Within each group, the scattered dots represent immune cells ssGSEA values. The thick line represents the median value. The bottom and top of the boxes are the 25th and 75th percentiles (interquartile range). The whiskers encompass 1.5 times the interquartile range. The statistical difference of three gene clusters was compared through the Kruskal–Wallis test. **b** CNE plot of top five GO pathways for differential expression genes. **c** KEGG results for differential expression genes between FCER1G high and low subgroups. The X-axis represents gene ratio and the Y-axis represents different enriched pathways. *P < 0.05; *P < 0.01; ***P < 0.001; ****P < 0.0001.

** Additional file 4: Figure S3.** Validation of correlation in TCGA cohort. **a** The correlation between PDCD1 and CD274. **b** The correlation between CXCR4 and CXCL12. **c** The correlation between CTLA4 and CD80. **d,e** The correlation between the expression of FCER1G and PDCD1 **d**, CTLA4 **e**

** Additional file 5: Figure S4.** Association between FCER1G expression and immunotherapeutic response. **a** Correlation between mRNA expression of FCER1G and PD1 in tumor tissues from glioma patients (n = 20). **b** SubMap analysis revealed that FCER1G-high subgroup could be more sensitive to immunotherapy (Bonferroni-corrected P value < 0.05). ROC curves for FCER1G in predicting the immunotherapy response of glioma patients. **c** CGGA, **d** TCGA.

** Additional file 6: Figure S5.**Quantify of immune cells and expression levels of immune check points in gliomas. **a**Quantify of immune cells between patients with different grades of glioma. **b** Expression levels of PDCD1 (PD1), CD274 (PDL1), and CTLA4 between different grades of glioma patients from CGGA and TCGA. **c** Kaplan-Meier plots of PDCD1, CD274, and CTLA4 in CGGA datasets. Patients were divided into high and low expressed group by the medium expression level. **d** Expression levels of PDCD1, CD274, and CTLA4 in FCER1G-high and FCER1G-low subgroup.

## Data Availability

Publicly available datasets were analyzed in this study. This data can be found here: http://gliovis.bioinfo.cnio.es/. The supplementary material for this article can be found online. All processed data and R codes used in this study can be obtained from the corresponding author on reasonable request.

## Abbreviations

CGGA: Chinese Glioma Genome Atlas
TCGA: The Cancer Genome Atlas
GEO: Gene Expression Omnibus
BBB: Blood-brain barrier
ICB: Immune checkpoint blockades
TIICs: Tumor infiltrating immune cells
FCER1G: High Affinity Immunoglobulin Epsilon Receptor Subunit Gamma
CTLA4: Cytotoxic T-lymphocyte associated protein 4
PD-1: Programmed cell death 1
DEG: Differential expressed gene
GO: Gene Ontology
BP: Biological process
MF: Molecular function
CC: Cellular component
GSEA: Gene Set Enrichment Analysis
KEGG: Kyoto Encyclopedia of Genes and Genomes
OS: Overall survival PFI progression-free interval
UVM: Uveal Melanoma
UCS: Uterine Carcinosarcoma
UCEC: Uterine Corpus Endometrial Carcinoma
THYM: Thymoma
THCA: Thyroid carcinoma
TGCT: Testicular Germ Cell Tumors
STAD: Stomach adenocarcinoma
SKCM: Skin Cutaneous Melanoma
SARC: Sarcoma
READ: Rectum adenocarcinoma
PRAD: Prostate adenocarcinoma
PCPG: Pheochromocytoma and Paraganglioma
PAAD: Pancreatic adenocarcinoma
OV: Ovarian serous cystadenocarcinoma
MESO: Mesothelioma
LUSC: Lung squamous cell carcinoma
LUAD: Lung adenocarcinoma
LIHC: Liver hepatocellular carcinoma
LGG: Brain Lower Grade Glioma
LCML: Chronic Myelogenous Leukemia
KIRP: Kidney renal papillary cell carcinoma
KIRC: Kidney renal clear cell carcinoma
KICH: Kidney Chromophobe
HNSC: Head and Neck squamous cell carcinoma
GBM: Glioblastoma multiforme
ESCA: Esophageal carcinoma
DLBC: Lymphoid Neoplasm Diffuse Large B-cell Lymphoma
COAD: Colon adenocarcinoma
CHOL: Cholangiocarcinoma
CESC: Cervical squamous cell carcinoma and endocervical adenocarcinoma
BRCA: Breast invasive carcinoma
BLCA: Bladder Urothelial Carcinoma
ACC: Adrenocortical carcinoma
