# Supplementary figures and images for: Prognostic and predictive value of FCER1G in glioma outcomes and response to immunotherapy
Source: Cancer Cell Int. 2021 Feb 12;21:103. doi: 10.1186/s12935-021-01804-3 (PMC7881595; doi:10.1186/s12935-021-01804-3)

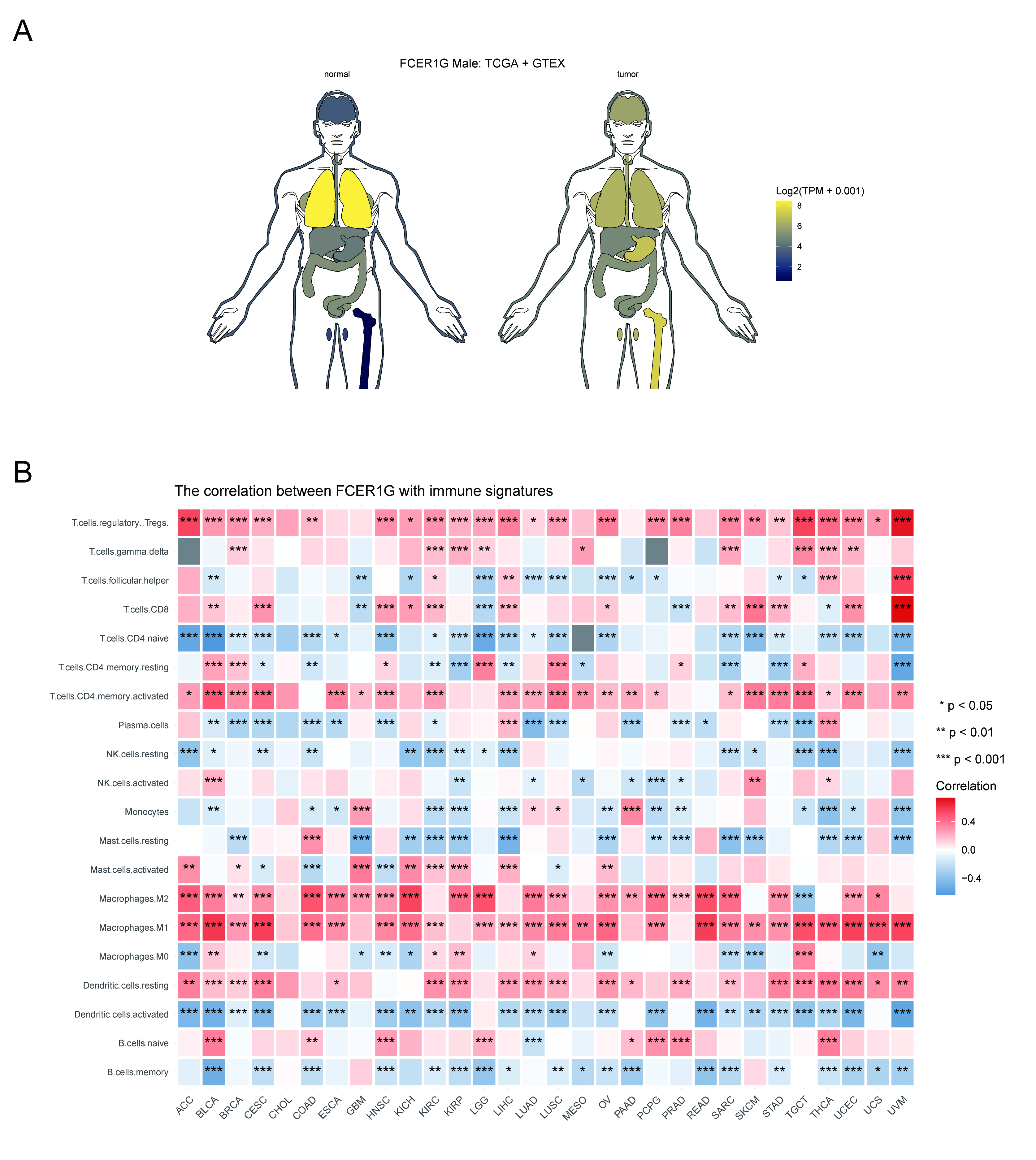

Supplement: Supplementary file 2 — Additional file 2: Figure S1. FCER1G expression and correlation with immune signatures in pan-cancer. a Anatomy graph shows significant differences in expression levels of FCER1G in various tumors and normal tissues. b FCER1G has a significant relationship with tumor microenvironment in various tumors. [file 12935_2021_1804_MOESM2_ESM.tif]

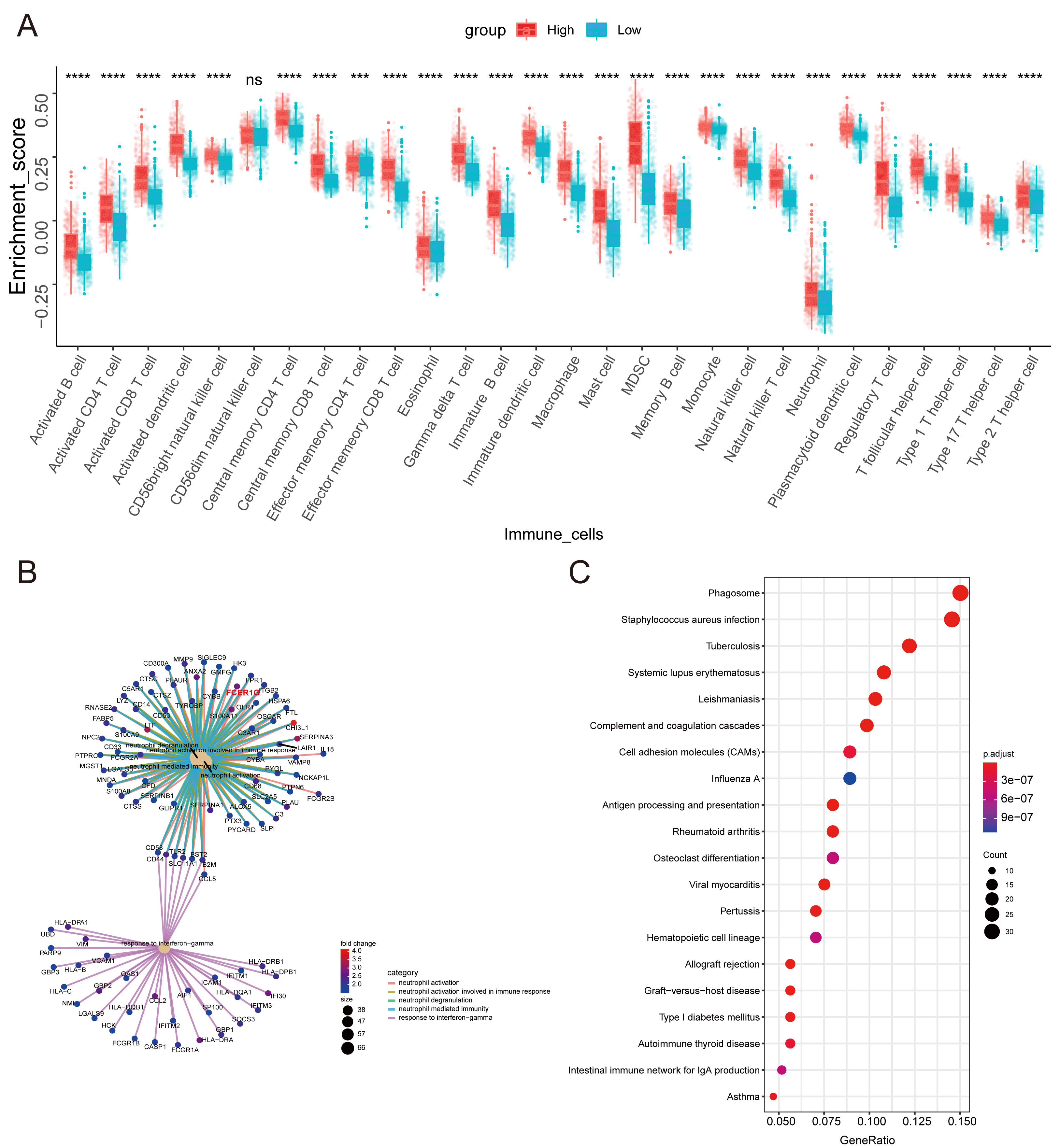

Supplement: Supplementary file 3 — Additional file 3: Figure S2. Immune cell fraction and GO analysis in FCER1G high and low subgroups. a The fraction of 28 immune cells in FCER1G high and low subgroups. Within each group, the scattered dots represent immune cells ssGSEA values. The thick line represents the median value. The bottom and top of the boxes are the 25th and 75th percentiles (interquartile range). The whiskers encompass 1.5 times the interquartile range. The statistical difference of three gene clusters was compared through the Kruskal–Wallis test. b CNE plot of top five GO pathways for differential expression genes. c KEGG results for differential expression genes between FCER1G high and low subgroups. The X-axis represents gene ratio and the Y-axis represents different enriched pathways. *P < 0.05; *P < 0.01; ***P < 0.001; ****P < 0.0001. [file 12935_2021_1804_MOESM3_ESM.tif]

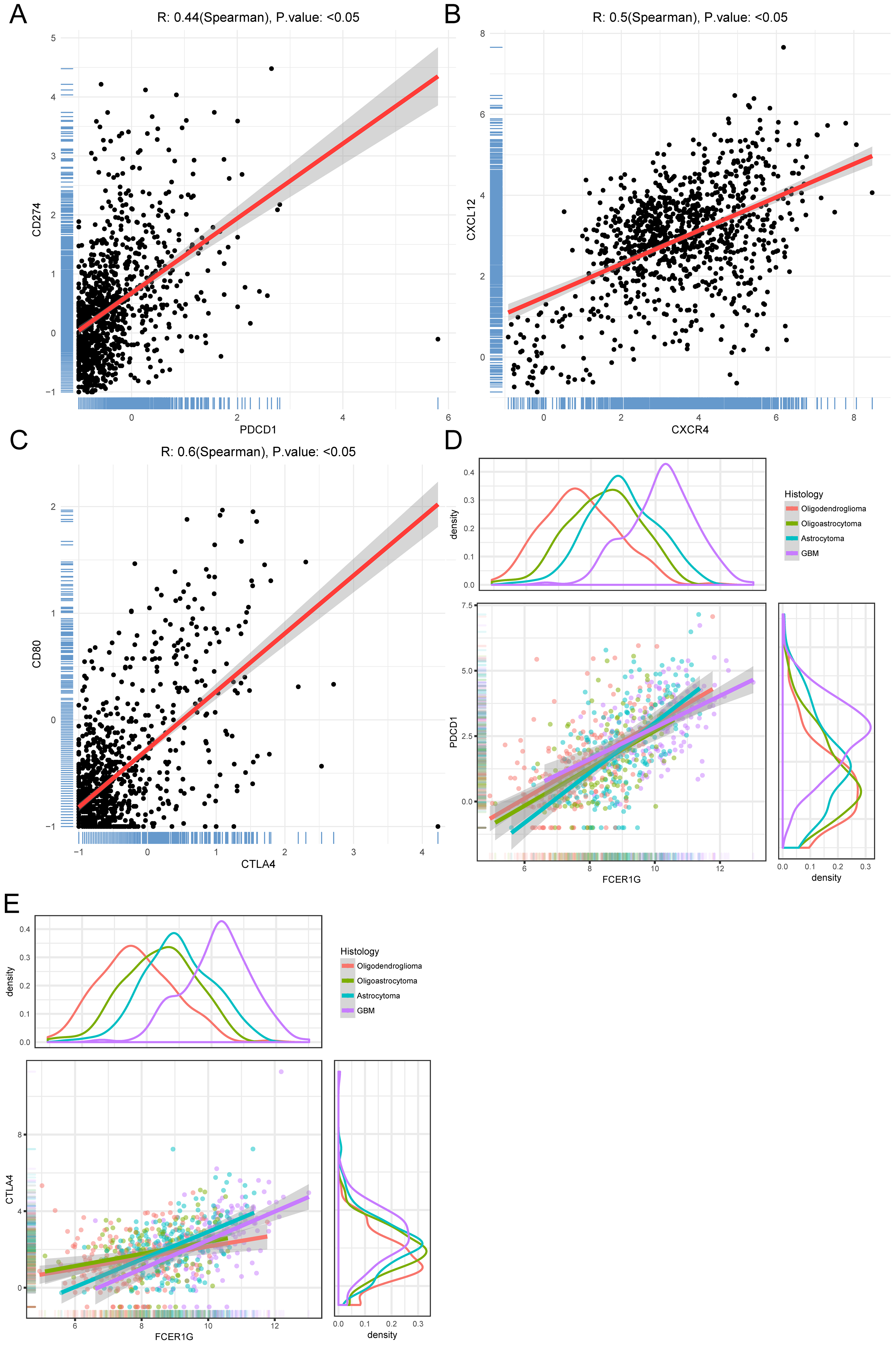

Supplement: Supplementary file 4 — Additional file 4: Figure S3. Validation of correlation in TCGA cohort. a The correlation between PDCD1 and CD274. b The correlation between CXCR4 and CXCL12. c The correlation between CTLA4 and CD80. d,e The correlation between the expression of FCER1G and PDCD1 d, CTLA4 e [file 12935_2021_1804_MOESM4_ESM.tif]

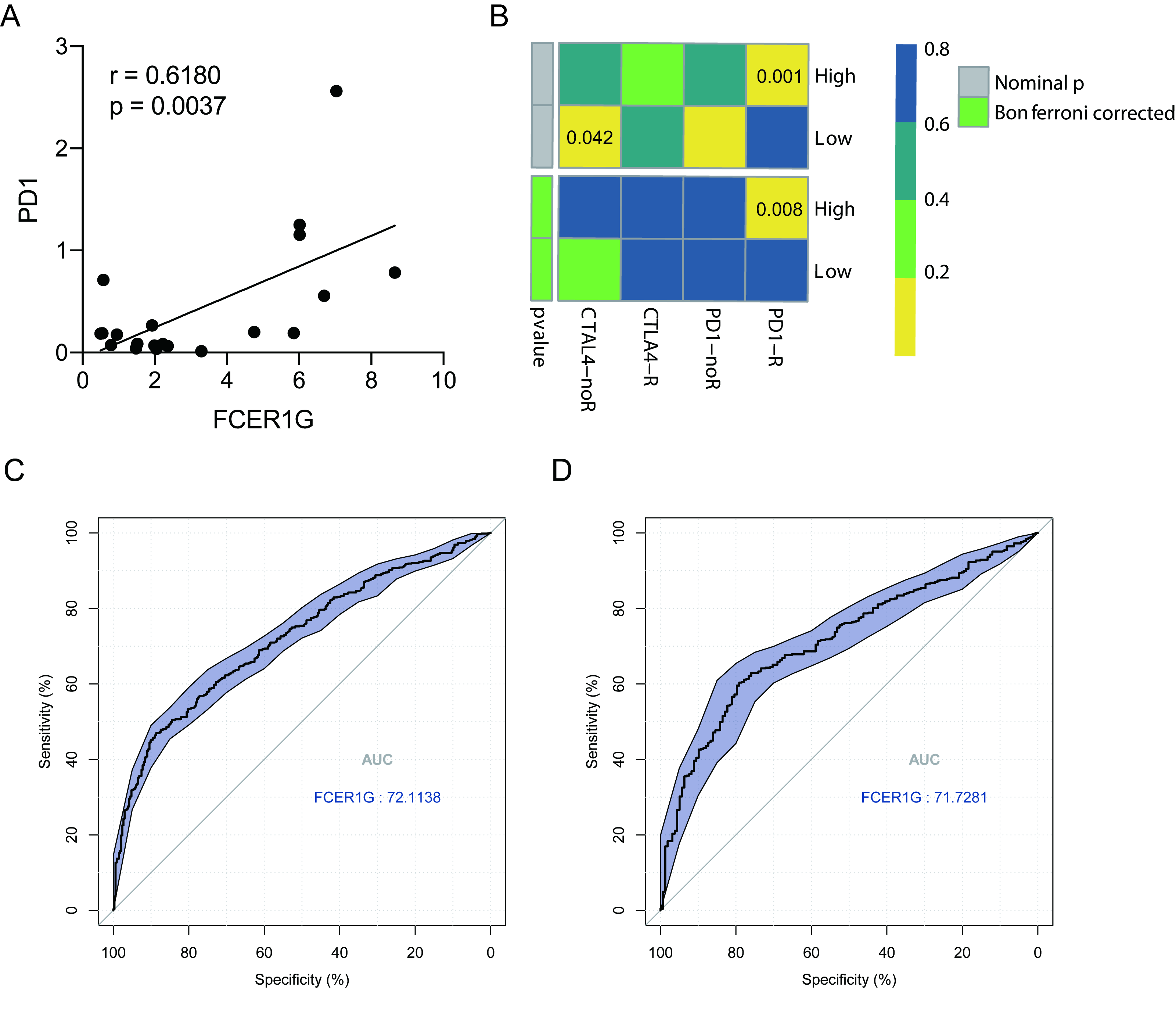

Supplement: Supplementary file 5 — Additional file 5: Figure S4. Association between FCER1G expression and immunotherapeutic response. a Correlation between mRNA expression of FCER1G and PD1 in tumor tissues from glioma patients (n = 20). b SubMap analysis revealed that FCER1G-high subgroup could be more sensitive to immunotherapy (Bonferroni-corrected P value < 0.05). ROC curves for FCER1G in predicting the immunotherapy response of glioma patients. c CGGA, d TCGA. [file 12935_2021_1804_MOESM5_ESM.tif]

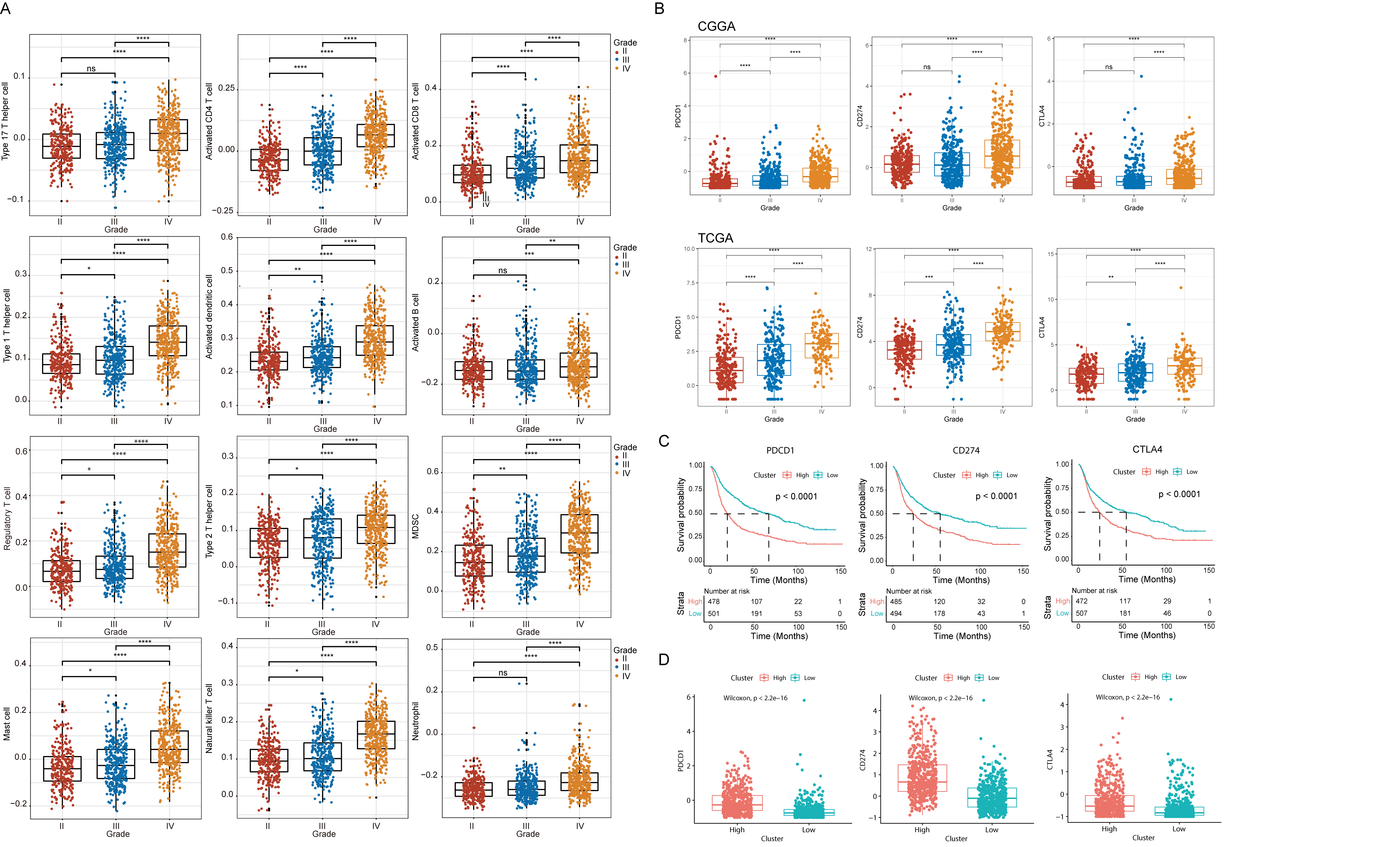

Supplement: Supplementary file 6 — Additional file 6: Figure S5.Quantify of immune cells and expression levels of immune check points in gliomas. aQuantify of immune cells between patients with different grades of glioma. b Expression levels of PDCD1 (PD1), CD274 (PDL1), and CTLA4 between different grades of glioma patients from CGGA and TCGA. c Kaplan-Meier plots of PDCD1, CD274, and CTLA4 in CGGA datasets. Patients were divided into high and low expressed group by the medium expression level. d Expression levels of PDCD1, CD274, and CTLA4 in FCER1G-high and FCER1G-low subgroup. [file 12935_2021_1804_MOESM6_ESM.tif]
